# Real-time deep learning-based model predictive control of a 3-DOF biped robot leg

**DOI:** 10.1038/s41598-024-66104-y

**Published:** 2024-07-15

**Authors:** Haitham El-Hussieny

**Affiliations:** Department of Mechatronics and Robotics Engineering, Egypt-Japan University of Science and Technology, E-JUST, Alexandria, 21934 Egypt

**Keywords:** Mechanical engineering, Electrical and electronic engineering

## Abstract

Our research utilized deep learning to enhance the control of a 3 Degrees of Freedom biped robot leg. We created a dynamic model based on a detailed joint angles and actuator torques dataset. This model was then integrated into a Model Predictive Control (MPC) framework, allowing for precise trajectory tracking without the need for traditional analytical dynamic models. By incorporating specific constraints within the MPC, we met operational and safety standards. The experimental results demonstrate the effectiveness of deep learning models in improving robotic control, leading to precise trajectory tracking and suggesting potential for further integration of deep learning into robotic system control. This approach not only outperforms traditional control methods in accuracy and efficiency but also opens the way for new research in robotics, highlighting the potential of utilizing deep learning models in predictive control techniques.

## Introduction

Robotic manipulation and movement describe how robotic systems engage with and transform their surroundings through meticulous control over their mechanical components^1^. The adoption of biped robots in real-time applications has seen a marked increase^2,3^, attributed primarily to their superior mobility compared to wheeled robots^4^. Given this context, extensive research over recent decades has aimed at refining the balanced walking abilities of biped robots^5^. The precision and efficiency of these robots’ movements are crucial^6^ because they fundamentally determine the robots’ reliability and performance in executing complex gaits, which necessitate advanced motor skills and sophisticated decision-making capabilities.

To achieve dynamically balanced gaits, it is essential to designate appropriate trajectories for the swing foot and hip joint of the biped robot^7^. Traditional approaches to robotic control have predominantly relied on analytical dynamic models, which are mathematical frameworks used to describe the physics of robot motion and interactions with the environment^8^. Model Predictive Control (MPC) stands out as a highly favored approach in gait control of biped robots because it integrates actuation and workspace limits and performance targets through an optimization framework^9,10^. The efficacy of MPC depends critically on having precise models of the system it controls. These models, which detail the robot’s kinematics and dynamics, are essential for creating control systems that are accurate and reliable, attributes vital for numerous robotic applications^11^. However, crafting predictive models often involves precise knowledge of physical parameters and environmental conditions, which may be hard to ascertain in real-world settings. Consequently, the accuracy of model development and parameter estimation becomes crucial, posing both a challenge and a necessity for the real-time deployment of these systems.

Accurate modeling presents unique challenges for biped robots due to the complexities introduced by high speeds and accelerations, which can lead to computationally demanding dynamic models^12^. The need for real-time control exacerbates these computational challenges. Dynamic models that handle complex interactions or multiple degrees of freedom often require solving computationally expensive differential equations. However, data-driven methods, particularly neural networks, have shown potential in accurately modeling these nonlinear dynamical effects without relying on exhaustive mathematical modeling^13,14^. Integrating such models as surrogates in MPC systems could facilitate meeting the real-time control requirements for legged and biped robots.

Previous studies utilizing predictive controllers have typically employed one of two approaches: using linear predictive models by linearizing the system around a fixed point^15^, or implementing gain scheduling to establish a multi-level controller where each level handles a specific operational mode^16^. While these methods can facilitate real-time operation, they tend to provide limited accuracy in predicting system responses. To improve prediction accuracy, some research has introduced nonlinear predictive models, such as in^17^. However, these models often fail to support real-time operation because solving the nonlinear equations involved requires extensive computation^18^. Meanwhile, alternative control strategies to MPC were employed. These strategies are characterized either by their non-predictive nature, as discussed in^19^, or by their avoidance of online optimization, as seen in the works by^20^ and^21^.

**Figure 1:**
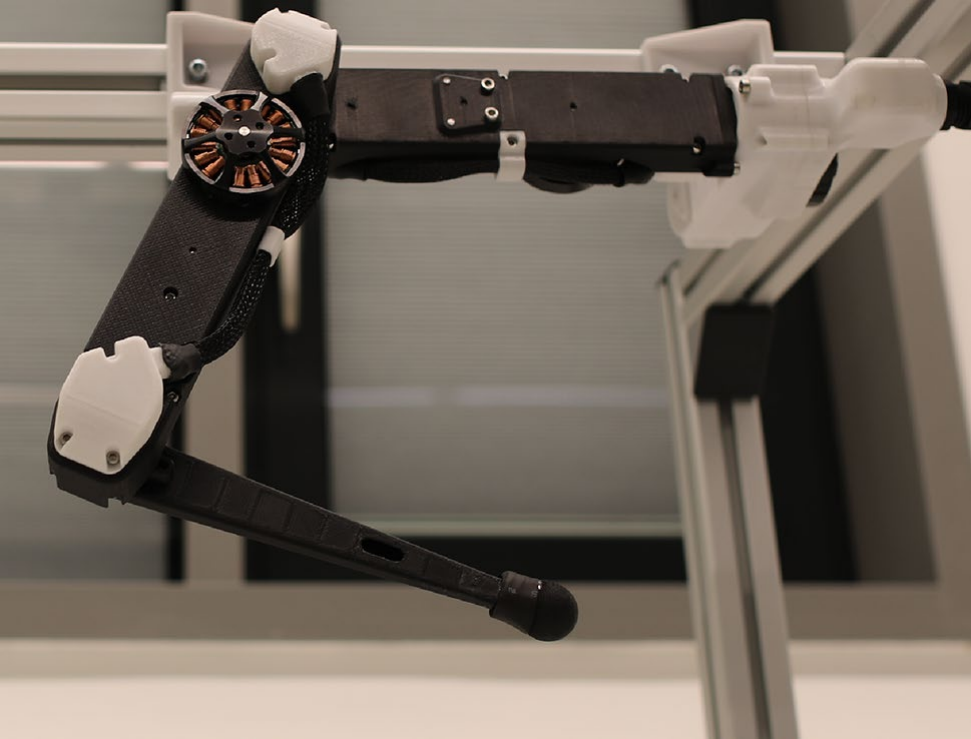
The three degree of freedom torque controlled robotic leg from Open Dynamic Robot Initiative^22^.

In this research, we introduce a dynamic model for a 3-degree-of-freedom (DOF) robotic leg, based on deep learning, as shown in Fig. 1. This model is subsequently incorporated into the MPC framework to improve trajectory tracking without the need for analytical dynamic models. We developed and validated the deep learning dynamic model using a comprehensive dataset of joint angles and actuator torques. Additionally, we conducted a theoretical analysis to ensure the stability and feasibility of the deep learning-based MPC. This model was successfully integrated into the MPC framework with additional constraints to enhance operational safety and efficiency. We also demonstrated experimentally the enhanced trajectory tracking capabilities and discussed the potential implications for future robotic control systems.

The organization of this paper is outlined as follows. Section "Deep Learning-based MPC" details the development of the deep learning-based Model Predictive Control (MPC) system. This section explains the construction of the data-driven dynamic model for the 3-degree-of-freedom (DOF) robotic leg, establishes the control objectives for the MPC, and provides an analysis of the system’s stability. Section "Results and Discussion" presents experimental results that demonstrate how the proposed deep learning-based MPC controls the robotic leg to accurately follow predefined joint values and trajectories while adhering to specific constraints. Finally, Section "Conclusion" concludes the paper and outlines potential directions for future research.

## Deep learning-based MPC

### Background

**Figure 2:**
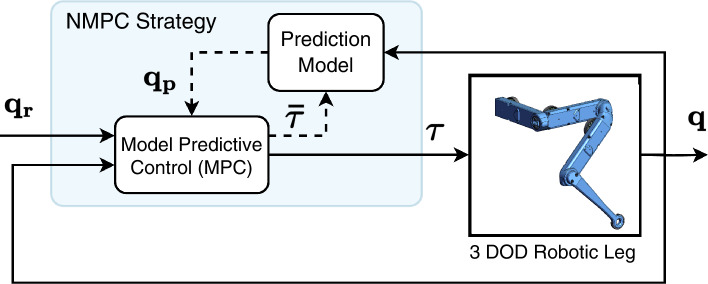
A block diagram illustrating the nonlinear model-predictive control (NMPC) strategy for controlling the 3 DOF robotic leg.

Model Predictive Control (MPC) is a method of multivariable control that utilizes a mathematical or data-driven model to forecast the future state of the system being controlled and calculates a series of optimal control inputs within specified constraints. At its core, NMPC comprises three key components: the predictive model, the target trajectory, and the controller that optimizes outcomes in a rolling fashion. The structure of a closed-loop NMPC system is illustrated in Fig. 2. In this figure, $\varvec{q}_r$ denotes the desired trajectory of the joint, $\varvec{\tau }$ refers to the torque variables being manipulated, $\varvec{q}$ is the controlled joint variable, and $\varvec{\bar{\tau }}$ signifies the sequence of torques that are optimized.

The control model for the three Degrees of Freedom (DOF) robotic leg can be expressed in discrete terms as follows:

1 $$\begin{aligned} \varvec{q}(k+1)=f\left( \varvec{q}(k),\varvec{\tau }(k)\right) \end{aligned}$$

wherein $\varvec{q} \in \mathbb {R}^3$ denotes the joint variable, $\varvec{\tau } \in \mathbb {R}^3$ represents the joint torques, and *f*(.) is the system’s unknown dynamic function. Given the complexity of the nonlinear system, finding a precise *f*(.) that accurately mirrors the robotic leg’s behavior could be challenging. Consequently, the primary aim of the introduced approach is to precisely forecast the system’s behavior under control and to derive optimal control actions. Thus, in this research we are utilizing a data-driven dynamics model that is used as the prediction model in the proposed MPC strategy.

### Data-driven dynamic model

The principal objective in the development of a Deep Neural Network (DNN) model is to construct a surrogate model that can be efficaciously employed as a predictive model within the framework of the proposed MPC strategy, aimed at regulating the joint variables of the robotic leg. More specifically, this endeavor seeks to ascertain an approximation for the joint variable $\varvec{q}$ at the subsequent time step $k+1$, predicated upon the existing joint variable $\varvec{q}$ and the applied input torque $\varvec{\tau }$ at the current time step *k*.Thus, the DNN is trained to directly approximate the solution in Eq. (1),

2 $$\begin{aligned} f\left( \varvec{q}(k),\varvec{\tau }(k); \varvec{\theta }\right) \approx f\left( \varvec{q}(k),\varvec{\tau }(k)\right) \end{aligned}$$

where $\varvec{\theta }$ are the free parameters of the DNN. This enhances the predictive capability and control precision within robotic leg systems.

**Figure 3:**
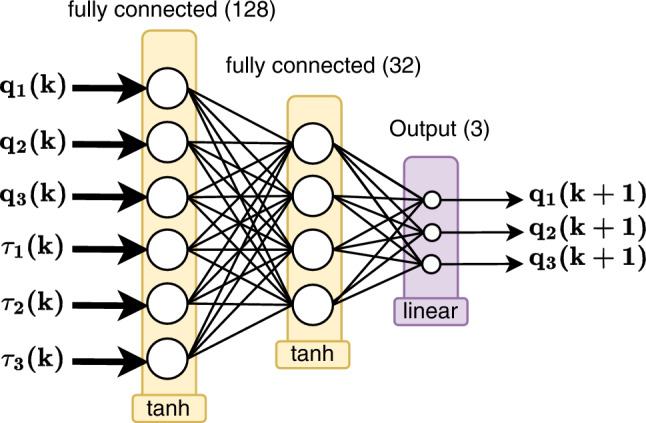
Architecture of the feed-forward neural network used for predicting joint values in a 3 DOF robotic leg.

A feed-forward shallow neural network depicted in Fig. 3 serves as the dynamic predictive model for the robotic leg. This network is structured to include an input layer, which accepts the current joint positions and input torques as its inputs. It features two hidden layers, with the first comprising 128 neurons and the second 32 neurons, both employing the hyperbolic tangent activation function. The architecture is completed by an output layer that uses a linear activation function, designed to predict the subsequent joint values. One potential future work could be testing Recurrent Neural Networks (RNN) and Long Short-Term Memory (LSTM) networks for building the deep learning model.

Dataset in^23^ were utilized, where captured from a 3 DoF torque-controlled real robotic leg, operating at a control and observation frequency of 1000 Hz, with sampling time of 1 ms. The system underwent multiple trials, each with a duration of 14 seconds, culminating 14000 samples. At each time index *t*, the control input comprised three joint torques $[\tau _1, \tau _2, \tau _3]^T$ (in Newton metres) dispatched to the motors at each joint. Observations, represented in a three-dimensional space, encompassed measured joint angles $[q_1, q_2, q_3]^T$ (in radians). Dataset are divided into 70% for training and 30% for validation.

In the development of our deep learning-based MPC, a critical requirement was the seamless integration and efficient execution of the predictive model within a diverse computational requirements. To this end, we leveraged the Open Neural Network Exchange (ONNX) framework^24^ for model conversion and interoperability. The original predictive model, designed and trained using TensorFlow API, demonstrated promising performance in initial experiments. The conversion process from TensorFlow to ONNX involved converting the TensorFlow computational graph, utilizing the tf2onnx tool, into an ONNX model file. This conversion process is streamlined and typically involves specifying the input and output nodes of the model to ensure the integrity of the model’s predictive capabilities post-conversion.

### Control objective

The primary goal of the proposed deep learning-based MPC is to ensure stabilization of the robotic leg, aiming for adherence to a predefined reference joint trajectory $\varvec{q}_r = [q_{1r}, q_{2r}, q_{3r}]^T$ in joint space. This objective encompasses the consideration of physical constraints, such as joint and actuation limits, during the determination of optimal control actions. Consequently, the cost function *J* is designed to assess both the tracking performance and the efficacy of control actions across a prediction horizon *N*, defined as:

3 $$\begin{aligned} \begin{aligned} \varvec{J}(k)&= \sum _{j=1}^{N} \varvec{e}^T_{(k+j)}\,\varvec{W}_1\,\varvec{e}_{(k+j)}\\&+ \sum _{j=1}^{N} \Delta \varvec{\tau }_{(k+j-1)}^T\,\varvec{W}_2\,\Delta \varvec{\tau }_{(k+j-1)} \end{aligned} \end{aligned}$$

here, $\varvec{e} = \varvec{q} - \varvec{q}_r$ represents the tracking error, and $\Delta \varvec{\tau }$ refers to the predicted increment in control input. The matrices $\varvec{W}_1 = w_1 \textbf{I}_3 \ge 0$ and $\varvec{W}_2 = w_2 \textbf{I}_3 \ge 0$ are weighting matrices, assumed to remain constant throughout the prediction horizon *N*.

The optimal control problem minimizing Eq. (3) is subject to the physical and actuators limits. The three joints in the robot’s are constrained between $[0, 2\pi ]$ defining the range of the angles, while the torques of the actuators are constrained according to the system limits between $[-2, +2]$

4 $$\begin{aligned} 0 \le \begin{bmatrix} q_1 (rad)\\ q_2 (rad)\\ q_3 (rad) \end{bmatrix}\le 2\pi , \qquad -2 \le \begin{bmatrix} \tau _1 (N.m)\\ \tau _2 (N.m)\\ \tau _3 (N.m) \end{bmatrix}\le +2 \end{aligned}$$

Moreover, nonlinear constraints in terms of the robot state, actuation, or parameters could be added for consideration while looking for the optimal control action:

5 $$\begin{aligned} \varvec{g}(\varvec{q}, \varvec{\tau }) \le 0 \end{aligned}$$

From the standpoint of supervised learning, the task of determining the optimal control law essentially represents a nonlinear mapping performed by a single-layer neural network^25^. Consequently, Gradient Descent (GD) emerges as a viable algorithm for this purpose. Accordingly, the sequence of control laws, $\varvec{\tau (k)}$, is updated as:

6 $$\begin{aligned} \varvec{\tau }(k+1)= & {} \varvec{\tau }(k) + \Delta \varvec{\tau }(k) \end{aligned}$$

7 $$\begin{aligned} \Delta \varvec{\tau }(k)= & {} \eta \left( - \dfrac{\partial \varvec{J}(k)}{\partial \varvec{\tau }}\right) \end{aligned}$$

where $\eta > 0$ represents the learning rate for the control sequence. As outlined in^26^, the control increment $\Delta \varvec{\tau }(k)$ is defined as

8 $$\begin{aligned} \Delta \varvec{\tau }(k) = \dfrac{\eta w_1}{1+\eta w_2} \left( \dfrac{\partial \varvec{q}}{\partial \varvec{\tau }} \right) ^T \varvec{e} \end{aligned}$$

In summary, Algorithm 1 details the procedure for two main tasks: First, it outlines the creation of the surrogate deep neural network (DNN) dynamic model for the 3-DOF (degrees of freedom) robotic leg, covered from line 1 to line 5. Second, it describes the application of a deep learning-based model predictive control (MPC) to accurately track the reference joint positions, which is discussed from line 6 to line 12.

**Algorithm 1:**
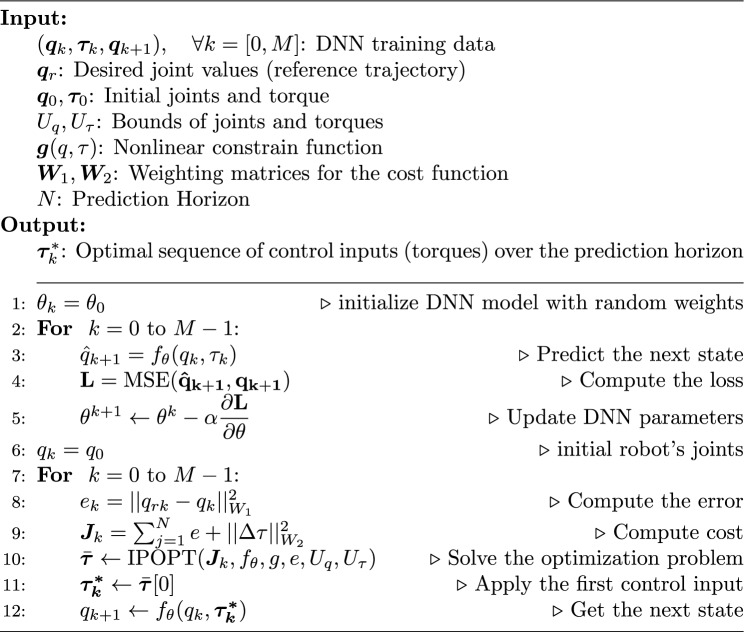
Deep Learning-Based MPC

### Stability analysis

To demonstrate the system’s stability, a quadratic Lyapunov function, defined in terms of the tracking error, is selected as follows:

9 $$\begin{aligned} V(\varvec{e}) = \frac{1}{2} \varvec{e}^T \varvec{e} \end{aligned}$$

where $\varvec{e} = \varvec{q}_r - \varvec{q}$. To guarantee global asymptotic stability of the system, the first time derivative of $V(\varvec{e})$ should be negative definite, indicating that $\varvec{e}$ exponentially converges to zero.

The first time derivative of $V(\varvec{e})$ is calculated as follows:

10 $$\begin{aligned} {\dot{V}}(\varvec{e}) = \varvec{e}^T \dot{\varvec{e}} \end{aligned}$$

Substituting the value of $\dot{\varvec{e}}$ into Eq. (10) yields:

11 $$\begin{aligned} \begin{aligned} {\dot{V}}(\varvec{e})&= \varvec{e}^T\left( \dot{\varvec{q}}_r - \dot{\varvec{q}}\right) \\&= \varvec{e}^T \left( \dfrac{\partial \varvec{q}_r}{\partial \varvec{\tau }} - \dfrac{\partial \varvec{q}}{\partial \varvec{\tau }} \right) \dfrac{\partial \varvec{\tau }}{\partial t}\\&=- \varvec{e}^T \left( \dfrac{\partial \varvec{q}}{\partial \varvec{\tau }} \right) \dfrac{\partial \varvec{\tau }}{\partial t}\\&\approxeq - \varvec{e}^T \left( \dfrac{\partial \varvec{q}}{\partial \varvec{\tau }} \right) \Delta \varvec{\tau }(k) \end{aligned} \end{aligned}$$

By substituting $\Delta \varvec{\tau }(k)$ in Eq. (8) into Eq. (11):

12 $$\begin{aligned} \begin{aligned} {\dot{V}}(\varvec{e})&= - \varvec{e}^T \left( \dfrac{\partial \varvec{q}}{\partial \varvec{\tau }} \right) \left\{ \dfrac{\eta w_1}{1+\eta w_2} \left( \dfrac{\partial \varvec{q}}{\partial \varvec{\tau }} \right) ^T \varvec{e}\right\} \\&= - \dfrac{\eta w_1}{1+\eta w_2} \left( \dfrac{\partial \varvec{q}}{\partial \varvec{\tau }} \right) ^T \left( \dfrac{\partial \varvec{q}}{\partial \varvec{\tau }} \right) \varvec{e}^T \varvec{e} \end{aligned} \end{aligned}$$

Given that ${\dot{V}}(\varvec{e}) < 0$, in accordance with Lyapunov stability theory, it can be concluded that the proposed control strategy is stable.

## Results and discussion

### Performance of the DNN prediction model

**Figure 4:**
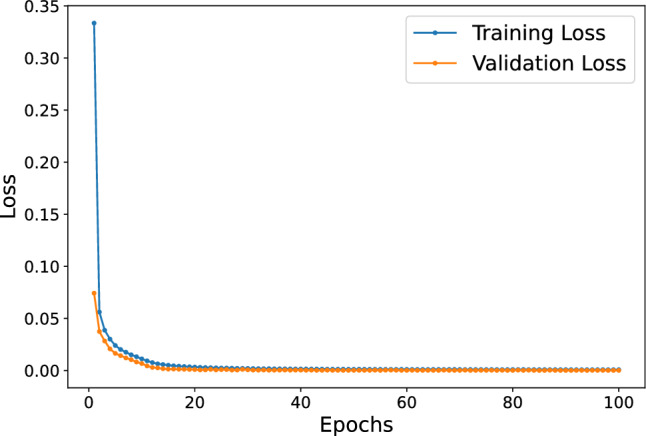
The loss graph showing the Mean Squared Error (MSE) of training and validation losses during the training of the DNN-based prediction over 100 epochs.

In our initial experiments, we investigated how well our DNN-based approach could model the movements of the 3 DOF robotic leg, using this model to predict future movements as part of the MPC system. We conducted both training and prediction tasks with the TensorFlow 2.x API on a standard desktop computer. The choice of computer hardware, especially the CPU’s clock speed of 2.8 GHz, significantly affected how long it took to train our model. Figure 4 shows the reduction in Mean Squared Error (MSE) losses for our DNN model over 100 training cycles, demonstrating a steady improvement in the model’s accuracy for both training and validation data sets.

**Figure 5:**
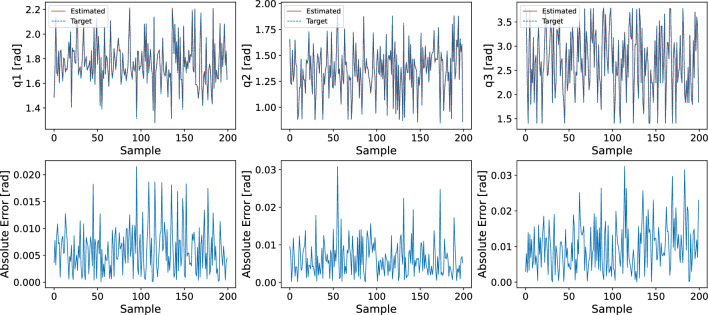
A comparison of target joints versus estimated joints using the proposed DNN-based prediction model.

To evaluate the effectiveness of our models, we tested them using a dataset containing 14,000 samples, processing these in groups of 128 at a time. In Fig. 5, we compare the predictions made by the DNN-based model against the actual values, focusing on the absolute difference between them. Due to constraints on space, we only display the results for the first 200 samples. The minimal discrepancy between the model’s predictions and the actual data is evident, as shown by the close match between the predicted outputs and the real target values.

### Time comparison with statics model

In this study, we compared the time taken by the DNN-based data-driven model and the statics model derived from Euler Lagrange formulation to solve the same problem of predicting the future joint angles given the current applied torques.

13 $$\begin{aligned} \frac{\textrm{d}}{\textrm{d}t}\frac{\partial {\mathcal {L}}}{\partial {\dot{q}}_i} - \frac{\partial {\mathcal {L}}}{\partial q_i} = \tau _i \end{aligned}$$

here, ${\mathcal {L}} = T - U$ represents the Lagrangian, which is the difference between the kinetic energy $T(\varvec{q},\dot{\varvec{q}})$ and the potential energy $U(\varvec{q})$. The term ${\tau _i}$ accounts for the applied torques acting on $q_i$. The model parameters such as links masses, inertias, and lengths are assumed with reasonable values estimated from the system CAD model.

The neural network model was trained using 14,000 samples, and the training process took 8.72 seconds. Once trained, the DNN-based model made predictions in just $0.01 \pm 0.002$ seconds per sample. In contrast, the mathematical statics model, formulated and solved using the ’ode45’ solver required $0.89 \pm 0.018$ seconds to find a solution. While the DNN-based model involves an initial training phase, its prediction phase is significantly faster, making it highly suitable for control applications requiring real-time decision-making. On the other hand, the mathematical model, though slower in execution, provides a direct analytical solution that may be more accurate under certain conditions.

### Point stabilization predictive control

**Figure 6:**
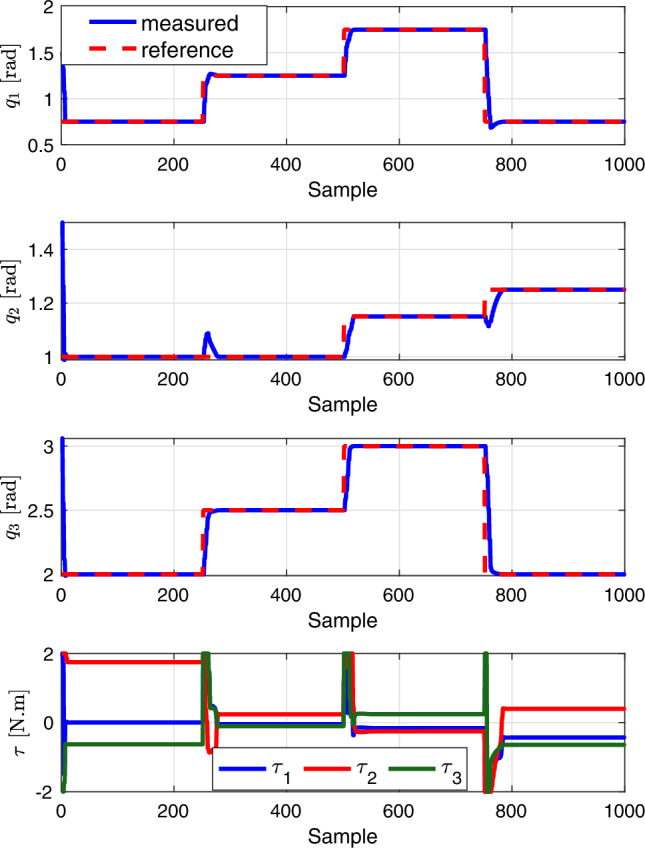
Results of point stabilization scenarios to evaluate the the proposed deep learning-based MPC for reaching predefined joints references.

In this series of experiments, we assessed the performance of a proposed deep learning-based MPC controller tailored for a robotic leg with 3 Degrees of Freedom (DOF), taking into account the constraints on its joints and inputs. This MPC controller was developed using the do-mpc framework^27^.

A key functionality of bipedal robots is their ability to move their legs to specific locations. Therefore, our initial experiment aimed at using the deep learning-based MPC controller to maintain the 3 DOF robotic leg’s joints at desired positions. Starting from an initial state of $\varvec{q}_0 =[1.5,1.5,3.06]^T$ (representing the leg’s joint angles) and $\varvec{\tau }_0 = [0, 0, 0]^T$ (representing the initial torques), the goal was to align the leg’s joints to predefined target positions, denoted by $\varvec{q}_r \in \mathbb {R}^3$. These target positions are indicative of the places the robot might need to navigate through during locomotion. The experiment was conducted with a sampling time of $T=0.001$ seconds and a prediction horizon of $N =20$. For the optimization cost function, the state and input weighting matrices were chosen to be diagonal, with $\varvec{W}_1 = \text {diag}(100, 100, 100)$ emphasizing the importance of accurately reaching the target joint positions, and $\varvec{W}_2 = \text {diag}(0.01, 0.01, 0.01)$ slightly penalizing input torques to achieve these positions.

Figure 6 illustrates the efficacy of our deep learning-based MPC controller in a point stabilization task. Initially, over the first 250 samples, the controller successfully maintains the robotic joints at a position of $(0.75, 1, 2)$ radians. Following this period, the target joint positions are sequentially updated to $(1.25, 1, 2.5)$, $(1.75, 1.15, 3)$, and $(0.75, 1.25, 2)$ for every subsequent 500, 750, and 1000 samples, respectively. The results demonstrate a significant reduction in tracking error, showcasing the controller’s capability to accurately achieve each set goal with the applied inputs.

Notably, during transitions to new target positions, a minor deviation in the joint position, specifically $\varvec{q}_2$, is observed. This deviation underscores the impact of the robot’s interconnected dynamics, which have been effectively incorporated into the DNN prediction model, indicating the model’s nuanced understanding of how changing one joint can influence others.

### Trajectory tracking predictive control

To evaluate the performance of the proposed deep learning-based MPC controller in trajectory tracking, we have utilized a reference trajectory. This trajectory is especially beneficial for situations where a robotic leg needs to adhere to a specific sequence of gaits of a biped robot across various terrains. Specifically, the reference trajectory, denoted as $q_r$, comprises a series of desired joint values. These values are sampled from Gaussian Processes (GPs), providing a well-defined set of samples for the desired motion.

**Figure 7:**
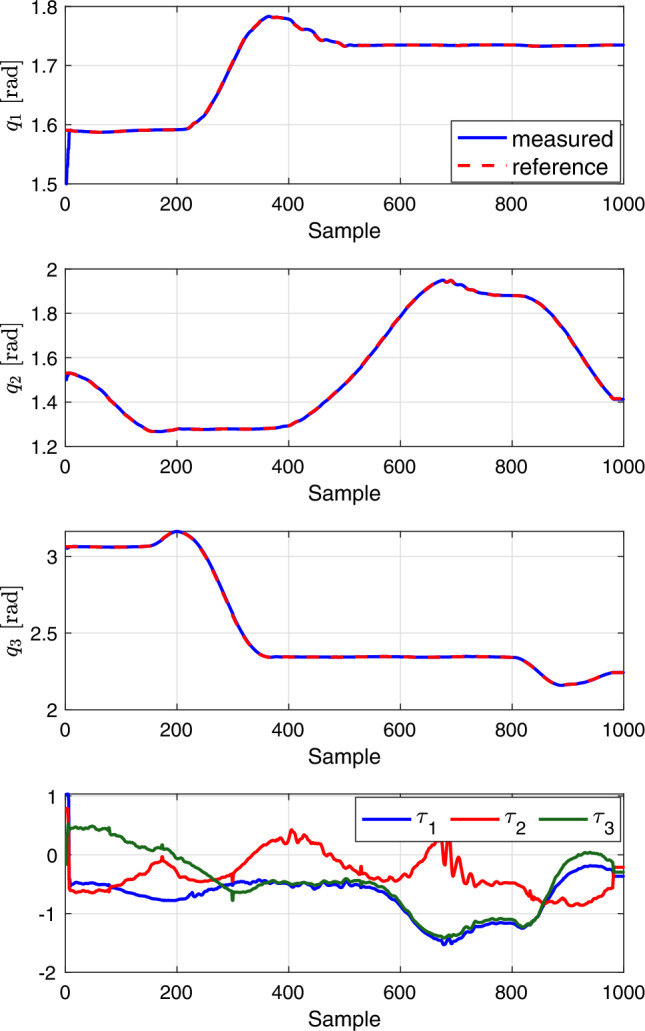
Results of trajectory tracking scenarios to evaluate the the proposed deep learning-based MPC for following joints reference trajectories.

**Figure 8:**
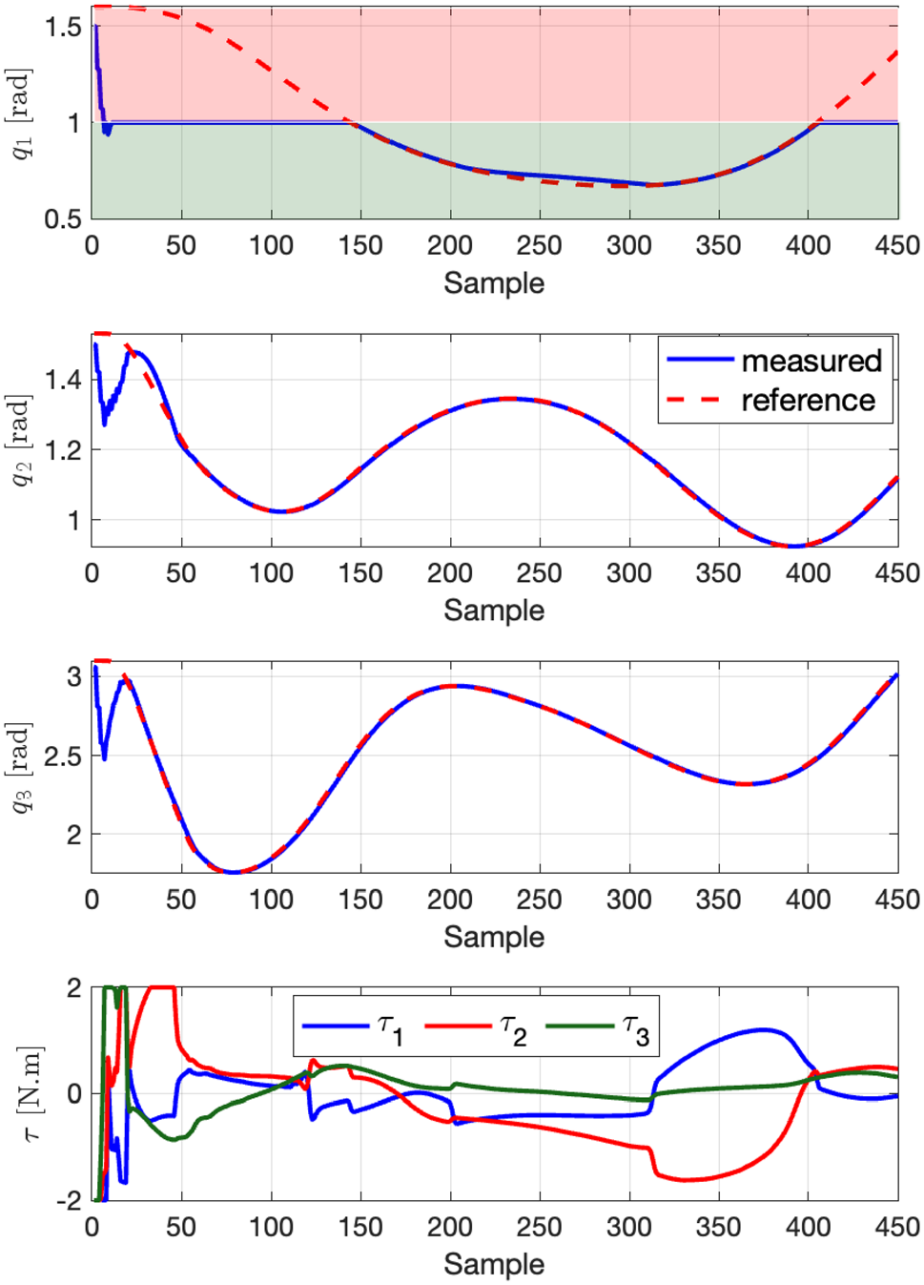
The result of the trajectory following experiment in the case of joint constraint on $q_1$ highlighted by the red area.

The robot begins with initial joint values denoted by $\varvec{q}_0 = [1.5, 1.5, 3.06]^T$. We have set the controller’s time step to $t=0.001$ seconds, and the prediction horizon is $N = 20$, across a total simulation time of 10 seconds. This setup utilizes the same values for $\varvec{W}_1$ and $\varvec{W}_2$ as those mentioned in the point stabilization experiment. The performance of the MPC is illustrated in Fig. 7, which highlights the effectiveness of the MPC in minimizing the discrepancy between the measured and the reference trajectories. The Mean Squared Error (MSE) between the reference trajectory and the actual robot trajectory is measured to be $2 \times 10^{-4}$ (in radians squared). Furthermore, Fig. 8 introduces an inequality nonlinear constraint on the robot’s joint $((q_1 - 1)^2 \le 0)$, serving as a limitation within the joint-space. Although the effect has not significantly appeared as a nonlinear constraint on the system itself, the concept has been tested within the algorithm. The results demonstrate that the proposed MPC is capable of achieving satisfactory tracking performance while adhering to this constraint, in addition to managing the constraints on the other joints effectively.

### Comparison with PID control

To assess how our deep learning-based MPC measures up against existing methods in scholarly works, we deployed a Proportional Integral Derivative (PID) control system. This system was utilized to direct a robotic leg to adhere to a predetermined trajectory. Within this PID controller, the torque $\tau _i$ for each joint is determined individually in the following manner:

14 $$\begin{aligned} \tau _i = K_p\ e_i + K_d \dfrac{de_i }{dt} + K_i \int e_i\ dt \end{aligned}$$

**Figure 9:**
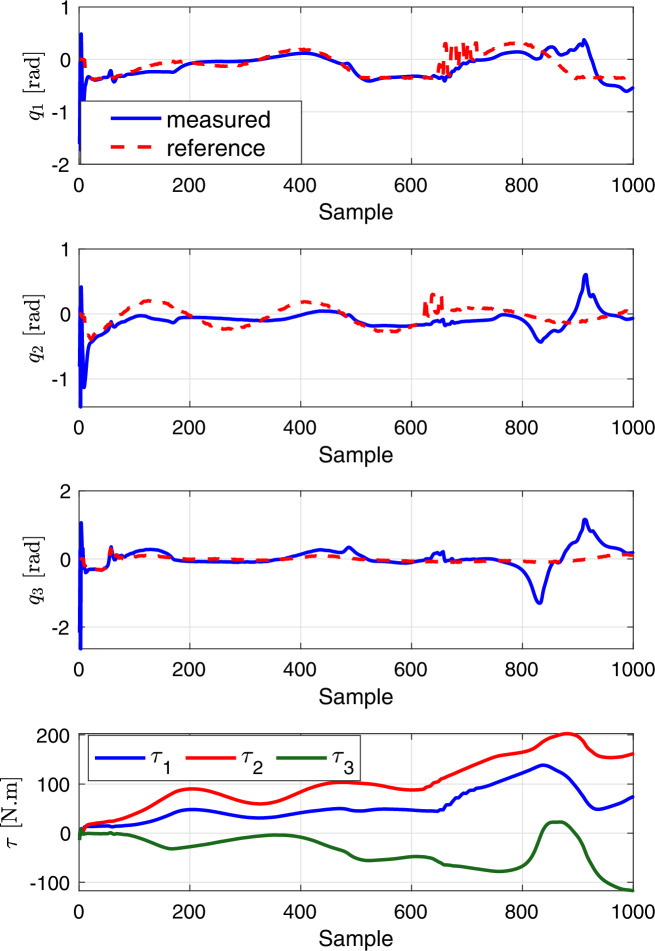
Performance of the PID tracking controller in following the reference joint trajectories.

In this context, $K_p = 0.1$, $K_i = 0.1$, and $K_d = 1.5$ represent the PID gains. The error $\varvec{e}_i = q_{ir} - q_i$ refers to the difference between the target trajectory of joint *i* and its actual position after torque application, as dictated by the Eq. (2), which describes the DNN dynamics model of the robotic leg. Figure 9 illustrates that the PID controller’s tracking capability is suboptimal, leading to the generation of excessive torques that breach the system’s limits. This underscores the effectiveness of our proposed predictive control strategy, which is capable of determining the minimal control efforts required to achieve the desired joint positions without violating the system’s constraints.

### DNN model selection

We carried out an extensive evaluation of the proposed DNN model for the robotic leg using the K-Fold Cross-Validation technique, following the method described by Anguita et al.^28^. This technique is essential for thoroughly assessing the model’s capability to predict the robot’s state under various architectural designs, ensuring both its effectiveness and reliability. We split the dataset into five distinct subsets to facilitate a cyclic process of training and evaluating the model. This comprehensive evaluation approach provides insights into the model’s performance under different conditions. Notably, this method helps identify the most effective architectural design and highlights the model’s ability to generalize to real-world scenarios.

In this study, we developed a suite of five deep neural DNN models that incorporate feed-forward architectures. These models vary in complexity, with trainable parameters ranging from 299 to 43,139. Each model consists of three layers, differentiated by the number of hidden units as detailed in Table 1. The layers are fully connected and marked with an ’F’ with a subscript denoting the count of neurons in each. The primary activation function used is the hyperbolic tangent (tanh), except for the output layer, which uses a linear activation function.

**Table 1:** Selected architectures for the 5-fold cross validation experiment.

Model	Hidden layers	Units	Avg. MSE $\times 10^{-5}$
1	$F_{16}, F_{8}, F_{4}$	299	64 $\pm 5.3$
2	$F_{32}, F_{16}, F_{8}$	915	37 $\pm 6.8$
3	$F_{64}, F_{32}, F_{16}$	3107	18 $\pm 1.4$
4	$F_{128}, F_{64}, F_{32}$	11331	9.2 $\pm 3.0$
5	$F_{256}, F_{128}, F_{64}$	43139	14 $\pm 7.1$

**Figure 10:**
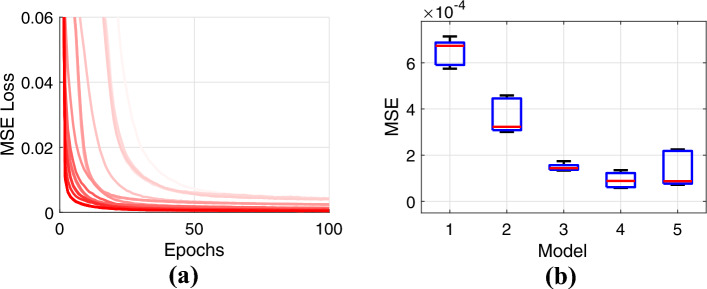
(**a**) Training losses expressed as mean squared error (MSE) for five deep neural network (DNN) models during 5-fold cross-validation with (**b**) mean and standard deviations.

These models underwent training over 100 epochs, utilizing the Adam optimizer with Mean Squared Error (MSE) as the primary loss metric. To enhance the thoroughness of evaluation and validation, a 5-fold cross-validation method was employed. The learning algorithm’s convergence trends, covering all network configurations and validation folds, are illustrated in Fig. 10a. Moreover, Fig. 10b offers an in-depth examination of the average MSE losses and their standard deviations across different model architectures. Significantly, networks featuring a higher parameter count are highlighted in dark red, signifying that more extensive networks tend to converge more effectively towards lower MSE values. For the sake of simplification in our selection methodology, we chose a neural network that demonstrated commendable performance throughout both training and testing stages. Investigating architectures that are both compact and capable of rapid learning offers potential to enhance the robustness of the overall system.

## Conclusion

To address the challenges of constrained nonlinear joint control for a three Degrees Of Freedom (DOF) robotic leg, this study introduces a new approach through the implementation of a deep learning-enhanced Nonlinear Model Predictive Control (MPC) method. Initially, a data-driven predictive model was developed, capable of forecasting future joint positions based on current joint values and applied torques. This model was then integrated within an MPC framework, employing an online optimization problem with process constraints to determine the optimal control torques for accurate joint trajectory tracking.

The efficacy of the proposed deep learning-based MPC joint control method was demonstrated through simulations across a variety of scenarios, including point stabilization, trajectory tracking, and constraints handling, all yielding satisfactory performance outcomes. Furthermore, a K-fold cross validation has been conducted to select the model architecture. The development of a Moving Horizon Estimation (MHE)^29^ presents a promising avenue for future research, potentially mitigating the current assumption of full state observability that this work presupposes. Such advancements promise to further refine the precision and applicability of MPC in robotic leg control, contributing valuable insights into the integration of deep learning techniques within complex control systems.

## Data Availability

The data that support the findings of this study are available from^23^ but restrictions apply to the availability of these data, which were used under license for the current study, and so are not publicly available. Data are however available from the first author upon reasonable request and with permission of^23^.

## References

[1] Siciliano, B., Khatib, O. & Kröger, T. *Springer Handbook of Robotics* Vol. 200 (Springer, 2008).

[2] Farid Y, Siciliano B, Ruggiero F (2022). Review and descriptive investigation of the connection between bipedal locomotion and non-prehensile manipulation. Annu. Rev. Control..

[3] Vukobratovic, M., Borovac, B., Surla, D. & Stokic, D. *Biped Locomotion: Dynamics, Stability, Control and Application* Vol. 7 (Springer, 2012).

[4] Collins SH, Wiggin MB, Sawicki GS (2015). Reducing the energy cost of human walking using an unpowered exoskeleton. Nature.

[5] Shigemi S, Goswami A, Vadakkepat P (2018). Asimo and humanoid robot research at honda. Hum. Robot.: Ref..

[6] Hopkins, M. A., Hong, D. W. & Leonessa, A. Humanoid locomotion on uneven terrain using the time-varying divergent component of motion. In *2014 IEEE-RAS International Conference on Humanoid Robots* 266–272 (IEEE, 2014).

[7] Chevallereau, C., Bessonnet, G., Abba, G. & Aoustin, Y. *Bipedal Robots: Modeling, Design and Walking Synthesis*. Wiley (2013).

[8] El-Hussieny, H. Dynamic modelling and task-space control of vine-like soft growing robots. In *2023 62nd Annual Conference of the Society of Instrument and Control Engineers (SICE)* 1220–1225 (IEEE, 2023).

[9] Schwenzer M, Ay M, Bergs T, Abel D (2021). Review on model predictive control: An engineering perspective. Int. J. Adv. Manuf. Technol..

[10] Katayama S, Murooka M, Tazaki Y (2023). Model predictive control of legged and humanoid robots: Models and algorithms. Adv. Robot..

[11] Wensing, P. M., Posa, M., Hu, Y., Escande, A., Mansard, N. & Del Prete, A. Optimization-based control for dynamic legged robots. *IEEE Trans. Robot.* (2023).

[12] Caux S, Zapata R (1999). Modeling and control of biped robot dynamics. Robotica.

[13] Salzmann T, Kaufmann E, Arrizabalaga J, Pavone M, Scaramuzza D, Ryll M (2023). Real-time neural mpc: Deep learning model predictive control for quadrotors and agile robotic platforms. IEEE Robot. Automat. Lett..

[14] El-Hussieny H, Hameed IA, Nada AA (2023). Deep CNN-based static modeling of soft robots utilizing absolute nodal coordinate formulation. Biomimetics.

[15] Wieber, P.-B. Trajectory free linear model predictive control for stable walking in the presence of strong perturbations. In *2006 6th IEEE-RAS International Conference on Humanoid Robots* 137–142 (IEEE, 2006).

[16] Li H, Frei RJ, Wensing PM (2021). Model hierarchy predictive control of robotic systems. IEEE Robot. Autom. Lett..

[17] García, G., Griffin, R. & Pratt, J. Mpc-based locomotion control of bipedal robots with line-feet contact using centroidal dynamics. In 2020 IEEE-RAS 20th International Conference on Humanoid Robots (Humanoids). IEEE, pp. 276–282 (2021).

[18] Ohtsuka, T. & Ozaki, K. Practical issues in nonlinear model predictive control: real-time optimization and systematic tuning. In *Nonlinear Model Predictive Control: Towards New Challenging Applications*, 447–460 (2009).

[19] Akbas, T., Eskimez, S. E., Ozel, S., Adak, O. K., Fidan, K. C. & Erbatur, K. Zero moment point based pace reference generation for quadruped robots via preview control. In *2012 12th IEEE International Workshop on Advanced Motion Control (AMC)*, pp. 1–7 (IEEE, 2012).

[20] Kolathaya S (2020). Local stability of pd controlled bipedal walking robots. Automatica.

[21] Sombolestan, M., Chen, Y. & Nguyen, Q. Adaptive force-based control for legged robots. In *2021 IEEE/RSJ International Conference on Intelligent Robots and Systems (IROS)* 7440–7447 (IEEE, 2021).

[22] Grimminger F, Meduri A, Khadiv M, Viereck J, Wüthrich M, Naveau M, Berenz V, Heim S, Widmaier F, Flayols T, Fiene J, Badri-Spröwitz A, Righetti L (2020). An open torque-controlled modular robot architecture for legged locomotion research. IEEE Robot. Autom. Lett..

[23] Agudelo-España, D., Zadaianchuk, A., Wenk, P., Garg, A., Akpo, J., Grimminger, F., Viereck, J., Naveau, M., Righetti, L., Martius, G., Krause, A., Schölkopf, B., Bauer, S. & Wüthrich, M. A real-robot dataset for assessing transferability of learned dynamics models. In *IEEE International Conference on Robotics and Automation (ICRA)* (2020).

[24] Bai, J., Lu, F., Zhang, K., *et al.* Onnx: Open neural network exchange. https://github.com/onnx/onnx (2019).

[25] Han H-G, Wu X-L, Qiao J-F (2013). Real-time model predictive control using a self-organizing neural network. IEEE Trans. Neural Networks Learn. Syst..

[26] Wang G, Jia Q-S, Qiao J, Bi J, Zhou M (2020). Deep learning-based model predictive control for continuous stirred-tank reactor system. IEEE Trans. Neural Networks Learn. Syst..

[27] Fiedler F, Karg B, Lüken L, Brandner D, Heinlein M, Brabender F, Lucia S (2023). DO-MPC: Towards fair nonlinear and robust model predictive control. Control. Eng. Pract..

[28] Anguita, D., Ghio, A., Ridella, S. & Sterpi, D. K-fold cross validation for error rate estimate in support vector machines. In DMIN, 291–297 (2009).

[29] El-Hussieny H, Hameed IA, Zaky AB (2023). Plant-inspired soft growing robots: A control approach using nonlinear model predictive techniques. Appl. Sci..

